# Maternal and perinatal health indicators in Brazil over a decade: assessing the impact of the COVID-19 pandemic and SARS-CoV-2 vaccination through interrupted time series analysis

**DOI:** 10.1016/j.lana.2024.100774

**Published:** 2024-05-23

**Authors:** Rita Carvalho-Sauer, Maria da Conceição Nascimento Costa, Maria Gloria Teixeira, Renzo Flores-Ortiz, Jessidenes Teixeira de Freitas Mendes Leal, Ramon Saavedra, Enny S. Paixao

**Affiliations:** a State Health Department of Bahia, Institute of Collective Health of the Federal University of Bahia, Brazil; b Institute of Collective Health of the Federal University of Bahia, Brazil; c Center for Data and Knowledge Integration for Health (CIDACS), Gonçalo Moniz Institute, Oswaldo Cruz Foundation (FIOCRUZ), Brazil; d Municipal Health Department of Salvador, Institute of Collective Health of the Federal University of Bahia, Brazil; e London School of Hygiene and Tropical Medicine, England

**Keywords:** COVID-19, SARS-CoV-2, Maternal mortality, Perinatal mortality, Cesarean section, Low birth weight, Premature birth, Apgar score, Respiratory distress syndrome, Health status indicators, Interrupted time series analysis

## Abstract

**Background:**

Few studies have evaluated the effects of the Coronavirus disease 2019 (COVID-19) pandemic, caused by the SARS-CoV-2 virus, on maternal and perinatal health at a populational level. We investigated maternal and perinatal health indicators in Brazil, focusing on the effects of the COVID-19 pandemic, and SARS-CoV-2 vaccination campaign for pregnant women.

**Methods:**

Utilizing interrupted time series analysis (January 2013–December 2022), we examined Maternal Mortality Ratio, Perinatal Mortality Rate, Preterm Birth Rate, Cesarean Section Rate, and other five indicators. Interruptions occurred at the pandemic's onset (March 2020) and pregnant women's vaccination (July 2021). Results were expressed as percent changes on time series' level and slope.

**Findings:**

The COVID-19 onset led to immediate spikes in Maternal Mortality Ratio (33.37%) and Perinatal Mortality Rate (3.20%) (p < 0.05). From March 2020 to December 2022, Cesarean Section and Preterm Birth Rates exhibited upward trends, growing monthly at 0.13% and 0.23%, respectively (p < 0.05). Post start of SARS-CoV-2 vaccination (July 2021), Maternal Mortality Ratio (−34.10%) and Cesarean Section Rate (−1.87%) promptly declined (p < 0.05). Subsequently, we observed a monthly decrease of Maternal Mortality Ratio (−9.43%) and increase of Cesarean Section Rate (0.25%) (p < 0.05), while Perinatal Mortality Rate and Preterm Birth Rate showed a stationary pattern.

**Interpretation:**

The pandemic worsened all analyzed health indicators. Despite improvements in Maternal Mortality Ratio, following the SARS-CoV-2 vaccination campaign for pregnant women, the other indicators continued to sustain altered patterns from the pre-pandemic period.

**Funding:**

No funding.

**Research in context**

**Evidence before this study**

Many studies have demonstrated the adverse impact of the SARS-CoV-2 virus on maternal and perinatal outcomes, as well as the disruption caused by the COVID-19 pandemic on access to and quality of prenatal care and childbirth. Socioeconomic factors have also been identified as influential in the observed outcomes of COVID-19 in pregnancy.

In this context, we searched for information on the possible effects of COVID-19 pandemic period on maternal and perinatal health indicators at the population-level. We used Google Scholar and PubMed to find articles in English, Spanish or Portuguese, which are the three most spoken languages on the American continent, with the terms ‘SARS-CoV-2’ OR ‘COVID-19 pandemic’, AND ‘maternal health indicators’, OR ‘perinatal health indicators’, OR ‘maternal mortality ratio’, OR ‘perinatal mortality rate’, OR ‘acute respiratory distress in pregnancy’ OR ‘cesarean section rate’ OR ‘facility birth rate’ OR ‘Apgar score’ OR ‘population based’, as of 2020.

We found that most studies on COVID-19 effects on maternal and perinatal health used a cohort or case–control design to compare pregnancy outcomes with and without exposure to SARS-CoV-2 infection. Few studies examined maternal and perinatal health indicators at a national populational level, before and during the COVID-19 pandemic. Those who did were mainly from rich countries. Studies on COVID-19 pandemic effects on these indicators in low- and middle-income countries are scarce.

**Added value of this study**

This study was conducted in Brazil, a middle-income country with 203 million inhabitants, seriously hit by COVID-19. We used reliable health information systems to analyze various maternal and perinatal health indicators (Maternal Mortality Ratio, Acute Respiratory Distress in Pregnancy Ratio, Perinatal Mortality Rate, Facility Births Rate, Cesarean Section Rate, Low Birth Weight Rate, Preterm Birth Rate, and Rate of Apgar score <7 at 1 and 5 min of live) before and after the COVID-19 pandemic onset, and the SARS-CoV-2 vaccination campaign.

We provide a comprehensive view of the COVID-19 pandemic's effects on maternal and perinatal health and discuss the challenges posed in achieving Sustainable Development Goals by 2030. Using a quasi-experimental method (interrupted time series analysis), we found that all studied indicators worsened by the COVID-19 pandemic from March 2020. The SARS-CoV-2 vaccination campaign for non-comorbidity pregnant women, that began in July 2021, improved maternal mortality and acute respiratory distress in pregnancy ratios and facility births rate. However, until December 2022, other studied indicators still showed a different trend from pre-pandemic patterns.

**Implications of all the available evidence**

Our findings reinforce knowledge about the damage caused by the COVID-19 pandemic on maternal and perinatal health outcomes. Moreover, the persistence of an altered pattern in maternal and perinatal health indicators in Brazil during the final stages of the COVID-19 public health emergency (December 2022), despite the successful SARS-CoV-2 vaccination campaign, suggests the presence of issues that go beyond the pandemic health crisis.

This highlights the urgent need for additional research and prompt interventions to enhance prenatal and childbirth care, address maternal and perinatal health's social determinants, mitigate socioeconomic disparities, and promote healthy pregnancy behaviors, aiming to enhance the quality of life of mothers and babies and achieve the 2030 agenda of the Sustainable Development Goals. It is advisable to examine these indicators nationally in other countries and within specific population subgroups.

## Introduction

Starting in 2020, the Coronavirus disease 2019 (COVID-19) pandemic, an acute infectious respiratory disease caused by the novel coronavirus SARS-CoV-2, has presented a confluence of sanitary and economic crises, posing unprecedented challenges to global healthcare systems. Its impact extends far beyond individuals infected with SARS-CoV-2, also affecting those requiring routine medical care.^1^

Early in this public health emergency, the implementation of social isolation measures and the fear of the SARS-CoV-2 virus led to a substantial decrease in elective medical appointments, including maternal and perinatal care services.^2^ As the incidence of COVID-19 surged, many healthcare facilities were compelled to prioritize the treatment of individuals presenting respiratory symptoms.^2^^,^^3^

The COVID-19 pandemic has considerably affected less wealthy countries.^4^ In Brazil, a middle-income country with a population of 203 million inhabitants (as of 2022), 36 million cases and 694 thousand deaths due to COVID-19 occurred between 2020 and 2022,^5^ placing a strain on its healthcare system. Additionally, Brazil's economy plunged, deepening existing social disparities.^6^

In this scenario, the maternal and child population has suffered significant impacts, including declining birth rates, reduced frequency of prenatal visits, increase of maternal mental health disorders, and a concerning rise in maternal mortality.^2^^,^^7^, ^8^, ^9^ However, few studies have examined the effects of the COVID-19 pandemic period on maternal and perinatal health at a populational level. The analysis of maternal and perinatal health indicators can provide objective measures of this effects, besides the living conditions, health, and well-being of mother-infant dyad, and help monitor, evaluate, and identify priority areas for public policies.^10^^,^^11^

Over the past few decades, Brazil has made progress in reducing infant mortality, which went from 47.1 per 1000 live births in 1990 to 13.3 in 2019,^11^ and maternal mortality from 143 per 100,000 live births in 1990 to 57.9 in 2019.^10^ Notwithstanding, numerous challenges persist in achieving the agreed-upon goals for 2030 Agenda as the Sustainable Development Goals (2016–2030),^10^, ^11^, ^12^ an international commitment to which Brazil is signatory; and the COVID-19 pandemic represented a threat to the strides made in maternal and child health.

In May 2023, the World Health Organization (WHO) declared the end of the Public Health Emergency of International Concern related to COVID-19.^13^ However, the complete recovery from its detrimental effects on specific groups’ health demands proactive efforts from countries. Evaluating the impact of the COVID-19 pandemic on maternal and perinatal health indicators can help identify effective mitigation strategies. This study aims to analyze the temporal evolution of maternal and perinatal health indicators in Brazil, with a specific focus on assessing the effects of the COVID-19 pandemic, including the periods before and after vaccination of pregnant women against SARS-CoV-2.

## Methods

### Study design, setting, population, and period

We conducted an interrupted time series (ITS) study to assess the effects of the COVID-19 pandemic and the SARS-CoV-2 vaccination campaign (exposures) on maternal and perinatal health indicators (outcomes) in Brazil, using national health information system's data from January 2013 to December 2022.

In Interrupted Time Series (ITS) studies, we assess the effect of an intervention on a time series of an outcome of interest at the population level.^14^ The intervention is introduced at a well-defined point in time, thus dividing the study period into pre- and post-intervention periods.^14^ To evaluate the effect of the intervention, the trend in the pre-intervention period is used as a control, capturing what would have happened without the intervention.^15^^,^^16^ Therefore, the assessment of the intervention's effect involves examining any statistically significant changes that occur in the post-intervention period, compared to the pre-intervention period.^14^, ^15^, ^16^

### Procedures

We used data from three Brazilian health information systems, the Live Birth Information System (*Sistema de Informação sobre Nascidos Vivos - SINASC*), the Mortality Information System (*Sistema de Informação sobre Mortalidade–SIM*), and the Acute Respiratory Syndrome Surveillance System (*Sistema de Vigilância Epidemiológica da Síndrome Respiratória Aguda Grave–SIVEP*).^17^^,^^18^ These information systems contain robust and reliable data on births, deaths, and cases of acute respiratory syndrome, which are subject to universal surveillance and mandatory reporting throughout Brazil.^17^ All records of births; fetal (≥20 gestational weeks), early neonatal (<7 days of life) and maternal deaths; and cases of acute respiratory distress in pregnant women were eligible. The data was collected in July 2023.

### Outcomes

We analyzed monthly time series of nine indicators (outcomes) related to maternal and perinatal health. We consider 1) maternal mortality ratio (per 100,000 live births) and 2) perinatal mortality rate (per 1000 births) as the main outcomes. The secondary outcomes were 3) acute respiratory distress in pregnancy ratio (per 100,000 live births); 4) facility births rate (%); 5) cesarean section rate (%); 6) low birth weight rate (%); 7) preterm birth rate (%); 8) Apgar score <7 at 1 min of life rate (%); and 9) Apgar score <7 at 5 min of life rate (%). The sources and methodology for calculating these indicators is described in Supplementary Table S1.

The definitions of Maternal death, Acute respiratory distress (ARD) during pregnancy, Perinatal death, Facility Birth, Live birth, Low birth weight, Preterm newborn and Apgar Score are described in Supplementary Table S2. To conduct and report the results of this observational study, we adhered to the STROBE initiative (Strobe checklist). We make available, for consultation, the values of the indicators on an annual basis, for Brazil and its major regions: North, Northeast, Southeast, South, and Central-West, in Supplementary Table S3.

Missing data accounted for 0.00% for the variable maternal mortality ratio; 0.00% for the acute respiratory distress in pregnancy ratio; 0.00% for the perinatal mortality rate; 0.01% for the facility births rate; 0.09% for the cesarean section rate; 0.04% for the low birth weight rate; 1.93% for the preterm birth rate; 2.09% for Apgar score <7 at 1 min of life; and 2.08% for Apgar score <7 at 5 min of life. The aggregated data used in this study are publicly available and freely accessible. Therefore, according to resolution n. 466/2012 of the National Health Council, approval by a research ethics committee is not required. Nevertheless, we adhered to all ethical principles outlined in the Declaration of Helsinki and complied with Brazilian regulations regarding research involving human beings.

### Statistical analyses

We used interrupted time series analysis^15^^,^^16^ to assess the effects of the COVID-19 pandemic and the SARS-CoV-2 vaccination campaign on the selected maternal-perinatal health indicators. In the analysis to assess the effects of the COVID-19 pandemic, the indicators' time series ranged from January 2013 to December 2022, with March 2020 considered the interruption time-point (as the first case of COVID-19 in Brazil was confirmed on February 26, 2020^19^). In the analysis to assess the effects of the SARS-CoV-2 vaccination campaign, the indicators’ time series ranged from March 2020 to December 2022, with July 2021 considered the interruption time-point (as it marks the start of the SARS-CoV-2 vaccination campaign among pregnant women without comorbidities in Brazil^20^).

Interrupted time series analysis was performed fitting a Prais-Winsten regression model, based on the following equation: Y_t_ = β0 + β1∗*Time* + β2∗*Intervention* + β3∗*Time after intervention*, where Y_t_ is the outcome (Each of the maternal-perinatal health indicators selected for this study) at time t; *Time* is a numeric variable indicating time in months; *Intervention* is a dummy variable indicating the pre-intervention period (coded 0) or the intervention period (coded 1) with the intervention being the COVID-19 pandemic or the SARS-CoV-2 vaccination campaign; β0 is the baseline level of the outcome; β1 is the change in outcome associated with a time unit increase in the pre-intervention period; β2 is the level change following the intervention; and β3 is the slope change following the intervention, compared with the trend before the intervention. The level expresses the initial value of each segment of the time series; and the slope refers to the inclination, which we will present in the form of percentage change of the values over the period covered by each segment. Statistical significance in the coefficients β2 and β3 indicates an immediate (change in level) and progressive (change in slope) effect of the intervention, respectively.^21^ This equation was applied once for each outcome variable (maternal mortality ratio; acute respiratory distress in pregnancy ratio; perinatal mortality rate; facility births rate; cesarean section rate; low birth weight rate; preterm birth rate; Apgar score <7 at 1 min of life rate; and Apgar score <7 at 5 min of life rate), with the interruption point being the start of the COVID-19 pandemic in Brazil (March 2020). And again with an interruption point at the start of the COVID-19 vaccination campaign for pregnant women (July 2021).

Prais-Winsten regression was used over Ordinary Least Squares regression to account for autocorrelation in the residuals.^15^^,^^21^ Autocorrelation in the residuals was assessed using the Durbin–Watson test. We reported the Durbin–Watson test statistic for the Prais-Winsten regression residuals. The Durbin–Watson test statistic ranges from 0 to 4, with a value of 2 indicating no autocorrelation in the residuals. Values below 2 suggest the presence of positive autocorrelation, and values above 2 indicate negative autocorrelation. The closer the value is to the extremes, the stronger the autocorrelation in the series.^21^

The logarithmic transformation was applied to all indicators to obtain regression coefficients that are interpreted as monthly percent change (MPC)^21^; also, this transformation contributes to mitigating heteroskedasticity. The MPC was reported along with its 95% confidence interval (CI).

Seasonal patterns were observed through the decomposition of the indicators' time series. Therefore, we included two pairs of sine and cosine functions of time^15^ in the above Prais-Winsten regression model to account for seasonality. The sine/cosine terms that were not statistically significant on a Student's T Test were dropped from the model.

The trends were classified as ‘increasing’ (if there is statistical evidence that the MPC value is > 0), ‘decreasing’ (if there is statistical evidence that the MPC value is < 0), or ‘stationary’ (if there is no statistical evidence that the MPC value is different from zero), after evaluating point estimates and confidence intervals.^19^

The level of statistical significance adopted for this study was 5%.

All analyses were conducted with STATA SE v. 15.1, and the graphs were drawn in Microsoft Power BI v. 2.119.323.0.

### Role of the funding source

Not applicable. This study does not have any source of funding.

## Results

In Brazil, in the seven years before the COVID-19 pandemic (January 2013–February 2020), the time series of Maternal Mortality Ratio (MPC: 0.08; 95% CI: −0.31, 0.53), Acute Respiratory Distress during pregnancy (MPC: 1.15; 95% CI: −0.31, 2.65), Facility Births Rate (MPC: −0.00; 95% CI: −0.00, 0.00) and Cesarean Section Rate (MPC: −0.06; 95% CI: −0.03, 0.02) were stationary; the indicators Perinatal Mortality Rate (MPC: −0.10; 95% CI: −0.13, −0.08), Preterm Birth Rate (MPC: −0.03; 95% CI: −0.06, −0.01) and, Apgar score <7 at the 1st minute rate (MPC: −0.14; 95% CI: −0.16, −0.12), and Apgar score <7 at the 5th minute rate (MPC: −0.18; 95% CI: −0.20, −0.16) had a decreasing trend; and the indicator Low birth weight rate (MPC: 0.04; 95% CI: 0.02, 0.05) had an increasing trend (Table 1).

**Table 1:** Interrupted time series analyses, with Prais-Winsten regression model, of maternal and perinatal health indicators over a decade: immediate and progressive impact of the COVID-19 pandemic.

Period	Pre-pandemic (January 2013–February 2020)		COVID-19 pandemic (March 2020–December 2022)				Durbin–Watson statistic^c^
Maternal and perinatal health indicators	Trend		Immediate impact^a^		Progressive impact^b^		
	Monthly percent change (95% CI)	Interpretation	Percent change (95% CI)	Interpretation	Monthly percent change (95% IC)	Interpretation	
Maternal mortality ratio (per 100,000 live births)	0.08 (−0.37, 0.53)	Stationary	**33.37 (2.93, 72.83)∗**	**Increase**	−1.24 (−2.83, 0.38)	Stationary	1.98
Acute respiratory distress in pregnancy (per 100,000 live births)	1.15 (−0.32, 2.65)	Stationary	**505.81 (165.78, 1280.88)∗∗∗**	**Increase**	−0.43 (−5.59, 4.99)	Stationary	1.55
Perinatal mortality rate (per 1000 births)	**−0.10 (−0.13, −0.08)∗∗∗**	**Decrease**	**3.20 (0.84, 5.63)∗∗**	**Increase**	0.09 (−0.01, 0.20)	Stationary	2.02
Facility births rate (%)	−0.00 (−0.00, 0.00)	Stationary	**−0.09 (−0.18, −0.01)∗**	**Decrease**	0.01 (−0.00, 0.01)	Stationary	2.10
Cesarean section rate (%)	−0.06 (−0.03, 0.02)	Stationary	0.50 (−1.10, 2.12)	No change	**0.13 (0.05, 0.21)∗∗**	**Increase**	2.44
Low birth weight rate (%)	**0.04 (0.02, 0.05)∗∗∗**	**Increase**	**−3.00 (−4.72, −1.25)∗∗∗**	**Decrease**	**0.37 (0.29, 0.45)∗∗∗**	**Increase**	1.95
Preterm birth rate (%)	**−0.03 (−0.06, −0.01)∗**	**Decrease**	1.81 (−0.91, 4.60)	No change	**0.23 (0.11, 0.35)∗∗∗**	**Increase**	1.96
Apgar score <7 at the 1st minute rate (%)	**−0.14 (−0.16, −0.12)∗∗∗**	**Decrease**	1.24 (−0.27, 2.76)	No change	**0.22 (0.16, 0.29)∗∗∗**	**Increase**	2.05
Apgar score <7 at the 5th minute rate (%)	**−0.18 (−0.20, −0.16)∗∗∗**	**Decrease**	0.51 (−1.50, 2.55)	No change	**0.30 (0.21, 0.39)∗∗∗**	**Increase**	2.01

95% CI, 95% Confidence Interval.

∗p < 0.05; ∗∗p < 0.01; ∗∗∗p < 0.001.

Results highlighted in bold presented a p-value lower than 0.05.

“No change” means that there was no statistical evidence of a level change following the segmentation of the series, compared to the preceding segment.

Brazil, January 2013–December 2022.

a Immediate impact represents change in level following the segmentation of the time series.

b Progressive impact indicates change in slope following the start of COVID-19 pandemic.

c Durbin Watson statistics after applying the Prais-Winsten model to correct for serial autocorrelation.

In March 2020, there was an increase in the level of the Maternal Mortality Ratio, Acute Respiratory Distress during pregnancy, and Perinatal Mortality, respectively, by 33.37%, 505.81% and 3.20%. Further, there was a reduction in the rates of Facility births (−0.09%; 95% CI: −0.18, −0.01) and Low Birth Weight (−3.00%; 95% CI: −4.72, −1.25) (Table 1).

**Fig. 1:**
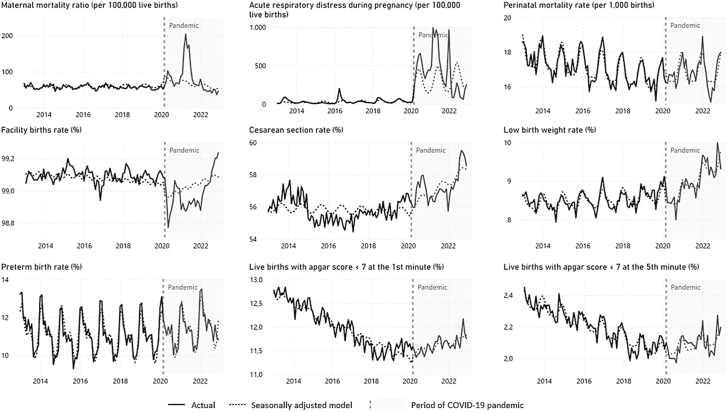
Interrupted time series analyses with Prais-Winsten regression model of maternal and perinatal health indicators over a decade. Brazil, Jan 2013 to Dec 2022. The models are adjusted for seasonality. Interruption applied on March 2020 (since the first COVID-19 case in Brazil was confirmed on Feb 26, 2020). The sources are Brazilian Ministry of Health - Live Birth Information System (SINASC), Mortality Information System (SIM), Acute Respiratory Syndrome Surveillance System (SIVEP). Data extracted on 07/14/2023. Data for 2022 are preliminary, subject to change.

During the COVID-19 pandemic period (March 2020–December 2022), the time series of the Maternal Mortality Ratio (MPC: −1.24; 95% CI: −2.83, 0.38), Acute Respiratory Distress in pregnancy (MPC: −0.43; 95% CI: −5.59, 4.99), Perinatal Mortality Rate (MPC: 0.09; 95% CI: −0.01, 0.20) and Facility Births Rate (MPC: −0.01; 95% CI: −0.00, 0.01) remained stationary, while the Cesarean Section Rate (MPC: 0.13; 95% CI: 0.05, 0.21), Low Birth Weight Rate (MPC: 0.37; 95% CI: 0.29, 0.45), Preterm Birth Rate (MPC: 0.23; 95% CI: 0.11, 0.35), and Apgar score <7 at the 1st minute rate (MPC: 0.22; 95% CI: 0.16, 0.29), and Apgar score <7 at the 5th minute rate (MPC: 0.30; 95% CI: 0.21, 0.39) minutes continued with a growing trend (Table 1). The graphic representation of the Interrupted Times Series of the indicators before and after the onset of the COVID-19 pandemic is shown in Fig. 1.

**Fig. 2:**
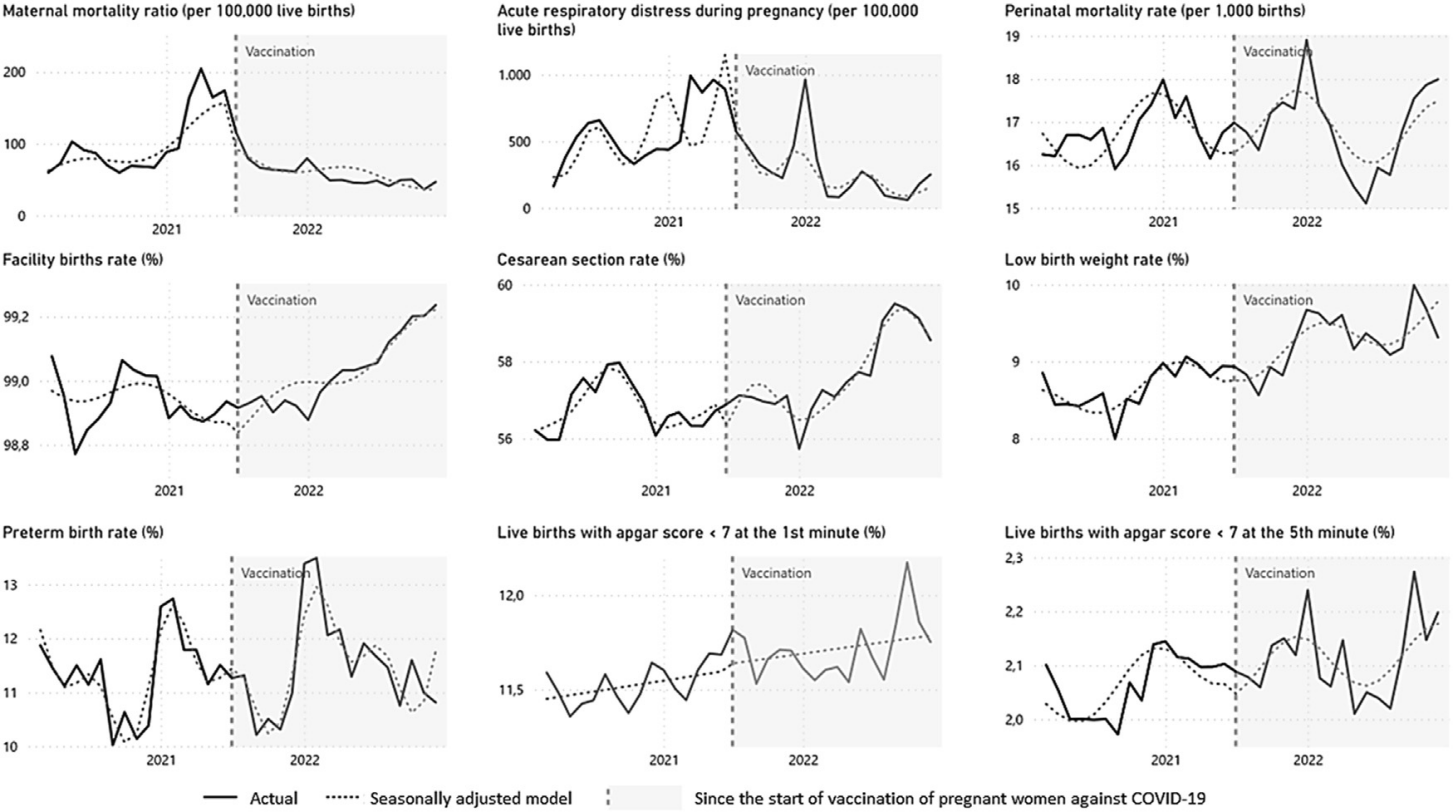
Interrupted time series analyses, with Prais-Winsten regression model, of maternal and perinatal health indicators during COVID-19 pandemic before and after the start of COVID-19 vaccination for pregnant women. Brazil, March 2020 to December 2022. Footnotes: The models are adjusted for seasonality. Interruption applied on July 2020 (when the Ministry of Health recommended the start of COVID-19 vaccination for pregnant women without comorbidities in Brazil). The sources are Brazilian Ministry of Health - Live Birth Information System (SINASC), Mortality Information System (SIM), Acute Respiratory Syndrome Surveillance System (SIVEP). Data extracted on 07/14/2023. Data for 2022 are preliminary, subject to change.

When specifically evaluating during the COVID-19 pandemic period (March 2020–December 2022), the possible effects of introducing vaccination against SARS-CoV-2 for pregnant women without comorbidities (July 2021), we identified a change in level of −34.10% in the Maternal Mortality Ratio and −1.87% in the Cesarean Section Rate, while the other indicators of the study did not show any statistically significant change in their level. Regarding the progressive impact (change in slope after intervention), there was a reduction in the Maternal Mortality Ratio (MPC: −9.43; 95% CI: −13.81, −4.72) and Acute Respiratory Distress during pregnancy (MPC: −13.19; 95% CI: −20.65, −5.03) and an increase in the Facility Births Rate (MPC: 0.03; 95% CI: 0.01, 0.04) and Cesarean Section Rate (MPC: 0.25; 95% CI: 0.16, 0.34); while the other indicators showed a stationarity pattern (Table 2). The graphic representation of the Interrupted Time Series of the indicators during the COVID-19 pandemic, before and after the start of vaccination against SARS-CoV-2 of pregnant women without comorbidities is shown in Fig. 2.

**Table 2:** Interrupted time series analyses, with Prais-Winsten regression model, of maternal and perinatal health indicators during COVID-19 pandemic: immediate and progressive impact of the COVID-19 vaccination for pregnant women.

Period	COVID-19 pandemic, before pregnant women vaccination (March 2020–June 2021)		COVID-19 pandemic, from the start of COVID-19 vaccination for pregnant women (July 2021–December 2022)				Durbin–Watson statistic^c^
Maternal and perinatal health indicators	Trend		Immediate impact^a^		Progressive impact^b^		
	Monthly percent change (95% CI)	Interpretation	Percent change (95% CI)	Interpretation	Monthly percent change (95% CI)	Interpretation	
Maternal mortality ratio (per 100,000 live births)	**5.86 (2.27, 9.57)∗∗**	**Increase**	**−34.10 (−54.10, −5.39)∗**	**Decrease**	**−9.43 (−13.81, −4.72)∗∗∗**	**Decrease**	1.82
Acute respiratory distress during pregnancy (per 100,000 live births)	5.95 (−0.66, 13.00)	Stationary	−38.16 (−70.50, 29.64)	No change	**−13.19 (−20.65, −5.03)∗∗**	**Decrease**	1.51
Perinatal mortality rate (per 1000 births)	−0.18 (−0.33, −0.69)	Stationary	−0.04 (−5.94, 6.24)	No change	−0.29 (−0.98, 0.41)	Stationary	1.93
Facility births rate (%)	−0.01 (−0.02, 0.00)	Stationary	−0.07 (−0.18, 0.04)	No change	**0.03 (0.01, 0.04)∗∗∗**	**Increase**	1.64
Cesarean section rate (%)	0.03 (−0.04, 0.10)	Stationary	**−1.87 (−2.72, −1.01)∗∗∗**	**Decrease**	**0.25 (0.16, 0.34)∗∗∗**	**Increase**	2.06
Low birth weight rate (%)	**0.34 (0.10, 0.58)∗∗**	**Increase**	0.66 (−2.41, 3.83)	No change	0.09 (−0.22, 0.40)	Stationary	1.88
Preterm birth rate (%)	0.07 (−0.27, 0.42)	Stationary	−0.21 (−4.57, 4.35)	No change	0.24 (−0.20, 0.68)	Stationary	2.16
Apgar score <7 at the 1st minute rate (%)	0.08 (−0.07, 0.24)	Stationary	0.30 (−1.52, 2.15)	No change	−0.01 (−0.21, 0.20)	Stationary	1.72
Apgar score <7 at the 5th minute rate (%)	**0.28 (0.004, 0.56)∗**	**Increase**	−1.27 (−4.67, 2.25)	No change	−0.19 (−0.55, 0.18)	Stationary	1.93

95% CI, 95% Confidence Interval.

∗p < 0.05; ∗∗p < 0.01; ∗∗∗p < 0.001.

Results highlighted in bold presented a p-value lower than 0.05.

“No change” means that there was no statistical evidence of a level change following the segmentation of the series, compared to the preceding segment.

Brazil, March 2020–December 2022.

a Immediate impact represents change in level following the segmentation of the time series.

b Progressive impact indicates change in slope following the start of COVID-19 vaccination for pregnant women without comorbidities.

c Durbin Watson statistics after applying the Prais-Winsten model to correct for serial autocorrelation.

## Discussion

Our results revealed that the COVID-19 pandemic has led to a worsening in maternal and perinatal health indicators in Brazil and, despite improvements in Maternal Mortality Ratio, Acute Respiratory Distress in pregnancy, and Facility Births Rate, following the SARS-CoV-2 vaccination campaign for pregnant women without comorbidities, the other indicators continued to sustain altered patterns from the pre-pandemic period.

As immediate impacts of the COVID-19 pandemic, we observed increases in maternal mortality, perinatal mortality, and acute respiratory distress (ARD) during pregnancy, as well as a reduction in facility births. After increase in level, these indicators displayed a stationary trend until December 2022.

Additionally, an immediate reduction in the rate of low birth weight was observed. However, in the months following March 2020, this indicator began to exhibit a consistent upward trend. Moreover, other progressive impacts of the COVID-19 pandemic in Brazil included a growing trend in the cesarean section rate, preterm births, and Apgar score <7 at 1st and 5th minutes of life.

While the effects of the COVID-19 pandemic on maternal and perinatal health indicators are not uniform worldwide, various other countries and territories, apart from Brazil have reported setbacks such as an increase in the maternal mortality ratio,^22^ cesarean section rates^23^ and perinatal deaths^24^ after the onset of the COVID-19 pandemic. On the other hand, the growing trend of premature births observed in Brazil was not common in other countries^24^^,^^25^ and needs to be investigated.

The link between SARS-CoV-2 infection during pregnancy and adverse obstetric and perinatal outcomes is well-established.^26^ However, understanding the effects of the COVID-19 pandemic on maternal and perinatal health indicators at the population level requires consideration beyond infection cases.

First, it is noteworthy that the strain on the healthcare system in the face of the COVID-19 crisis may have resulted in barriers to accessing obstetric and perinatal care, compromising the quantity and quality of antenatal consultations and childbirth assistance, leading to worse outcomes for mothers and babies.^2^^,^^27^

Second, mobility restrictions during lockdown periods and the fear of contracting an infection while looking for healthcare may have discouraged many pregnant women from seeking specialized healthcare.^2^^,^^27^ Consequently, diagnoses and interventions may have been delayed or insufficient, impairing the prognosis of morbidities and obstetric complications,^2^^,^^27^ and even the choice of birthing location may have been influenced,^28^ as evidenced by the decline in the facility birth rate.

Lastly, social vulnerabilities that emerged or worsened in Brazil during the COVID-19 pandemic, such as hunger, unemployment, and family impoverishment,^6^ can influence the assessed health indicators, as many of them are sensitive to living conditions and social determinants of health.

In contrast, the decrease in low birth weight during the initial period of the pandemic was intriguing. This reduction was also observed in high income countries^29^ and can be partly attributed to pregnant women staying at home during early stages of lockdown, resulting in lower exposure to air pollution and another stressor, and changes in nutrition. Unfortunately, this level drop was transient as the trend of low birth weight rate became increasing as the COVID-19 pandemic progressed.

The introduction of SARS-CoV-2 vaccination for pregnant women without comorbidities in July 2021 was associated with an immediate and significant reduction in maternal mortality ratio and cesarean section rate. Undoubtedly, SARS-CoV-2 vaccination has proven to be the most effective tool in mitigating this virus.^30^ Although this reduction in maternal deaths and cesarean sections was promising, it is essential to also analyze the progressive impacts of vaccination, as its effects tend to become more evident as vaccine coverage increases.

In this regard, the subsequent decline in the Maternal Mortality Ratio and Acute Respiratory Distress during pregnancy Ratio, indicators directly influenced by SARS-CoV-2 infections, suggests a positive effect of vaccination, emphasizing the importance of immunization in protecting the health of pregnant women.^31^

In the months following the introduction of vaccination, the observed increase in facility births and cesarean sections may indicate a reduction in the healthcare system's burden and a restoration of pregnant women's confidence in accessing healthcare services, as well as potential changes in medical decision-making. In a meta-analysis, Marchand et al.^31^ found high rates of cesarean section in pregnant women vaccinated against COVID-19 (OR = 1.20 CI = 1.05, 1.38), but they could not identify a clear explanation for this finding. Silva, Guida, and Costa,^32^ in a study conducted in a hospital in the Southeast region of Brazil, analyzed potential reasons for the increase in cesarean section rates during the COVID-19 pandemic. They found that maternal requests more than doubled compared to the pre-pandemic period, surpassing other motivations such as fetal distress and repeated cesarean sections, and this may be due to the possible uncertainties and fears of pregnant women in a pandemic scenario. It is worth mentioning that the overuse of cesarean sections was already a problem in Brazil before the COVID-19 pandemic,^33^ and the increase in cesarean section rates during the pandemic, despite its benefits in certain cases, may reflect overmedicalization with risks to maternal and neonatal health.^34^

It is concerning that only the Maternal Mortality Ratio returned to pre-pandemic levels by December 2022. The stationary trend in the ARD during pregnancy ratio, perinatal mortality rate, and facility births, coupled with the increasing trajectory of cesarean section, low birth weight, preterm birth, and Apgar score <7 at 1st and 5th minutes rates, even after Brazil achieved high vaccination coverage among pregnant women and the general population, may suggest systemic issues that extend beyond the health crisis. This raises an urgent need for further investigation and timely interventions.

The interpretation of our results should consider the strengths and limitations of this study. The impact of the COVID-19 pandemic and the SARS-CoV-2 vaccination campaign on maternal and perinatal health indicators may vary across different countries and regions, population subgroups, as well as social, economic, and epidemiological settings. Furthermore, there are limitations inherent to the use of administrative data, whose quality can vary over time and between regions of Brazil,^35^ besides the possibility of underreporting and the use of preliminary data for the year 2022, which may be subject to change. Also, external factors with the potential for confounding were not included in the models.

However, the strengths are the utilization of nationwide databases of events under universal surveillance and compulsory notification, coming from robust and reliable information systems. Additionally, this study uses interrupted time series analysis, which is among the strongest quasi-experimental methods for evaluating population-level intervention effects,^14^^,^^15^ and our models were designed to deal with seasonality and long-term trends.

Finally, in our study, which evaluates nine outcome variables, we confront the challenge of multiplicity of statistical tests and the consequent risk of Type I error inflation, as there is an overall increase in the probability of finding a significant result by chance as the number of tests increases.^36^ Acknowledging this, we emphasize the importance of interpreting our results by taking into account the context, effect sizes, and confidence intervals, while also recognizing the exploratory nature of certain analyses. Our findings can contribute to highlight the need to prioritize strategies aligned with the 2030 Agenda for Sustainable Development Goals,^12^ for improving maternal and perinatal health. It is worrying to note that at the end of 2022 the rates of cesarean sections, preterm births, low birth weight and Apgar score <7 were on an upward trend, and that hospitalizations of pregnant and postpartum women with acute respiratory distress remained well above the historical average.

It is urgent to invest in continuous improvement of prenatal and childbirth care, promotion of evidence-based interventions, addressing social determinants of maternal and perinatal health, combating socio-economic inequalities, and encouraging healthy behaviors during pregnancy. Further studies are also recommended to assess the factors associated with the changes in these indicators, including stratified analysis by specific groups, such as ethnic minorities, low-income pregnant women, and other situations of social vulnerability.

## Contributors

RCS, MCNC, and ESP conceptualized the study. RCS and RS collected data. RCS, RFO and JTFML analyzed the data. RCS, MCNC, MGT, RFO, JTFML, RS and ESP interpreted and discussed the results. All authors reviewed and approved the manuscript for publication.

## Data sharing statement

The data used in this study are in the public domain and available on the website of the Brazilian Ministry of Health at: <https://datasus.saude.gov.br/transferencia-de-arquivos/>; and <https://opendatasus.saude.gov.br/dataset?tags=SRAG>. The authors will also make the data available upon request.

## Declaration of interests

All authors declare that they have no conflicts of interest.
