# Supplementary material for: Maternal and perinatal health indicators in Brazil over a decade: assessing the impact of the COVID-19 pandemic and SARS-CoV-2 vaccination through interrupted time series analysis
Source: Lancet Reg Health Am. 2024 May 23;35:100774. doi: 10.1016/j.lana.2024.100774 (PMC11143910; doi:10.1016/j.lana.2024.100774)
Supplement: Supplementary material [file mmc1.docx]

**Maternal and Perinatal Health Indicators in Brazil over a decade: Assessing the Impact of the COVID-19 Pandemic and SARS-CoV-2 Vaccination through Interrupted Time Series Analysis**.

**Supplementary material**

| **Description** | **Page number** |
| --- | --- |
| **Supplementary Table 1.** Calculation method of health indicators selected for the study “Maternal and Perinatal Health Indicators in Brazil over a decade: Assessing the Impact of the COVID-19 Pandemic and SARS-CoV-2 Vaccination through Interrupted Time Series Analysis”. | 2 |
| **Supplementary Table 2.** Terminologies and definitions adopted for the study “Maternal and Perinatal Health Indicators in Brazil over a decade: Assessing the Impact of the COVID-19 Pandemic and SARS-CoV-2 Vaccination through Interrupted Time Series Analysis”. | 3 |
| **Supplementary Table 3.** Maternal and perinatal health indicators, according to year of occurrence. Brazil and major regions (North, Northeast, Southeast, South, Central-West), 2013-2022. | 4 |
| **STROBE checklist** for the study titled “Maternal and Perinatal Health Indicators in Brazil over a decade: Assessing the Impact of the COVID-19 Pandemic and SARS-CoV-2 Vaccination through Interrupted Time Series Analysis”. | 7 |

**Supplementary Table 1.** Calculation method of health indicators selected for the study “Maternal and Perinatal Health Indicators in Brazil over a decade: Assessing the Impact of the COVID-19 Pandemic and SARS-CoV-2 Vaccination through Interrupted Time Series Analysis”.

| **Health Indicator** | **Calculation method ^a^** | | **Source of data ^b^** |
| --- | --- | --- | --- |
|  | **Numerator** | **Denominator** |  |
| **Maternal Mortality Ratio (per 100,000 live births)** | Number of maternal deaths | Number of live births x 100,000 | SIM;  SINASC |
| **Acute Respiratory Distress during Pregnancy Ratio (per 100,000 live births)** | Number of cases of acute respiratory distress during pregnancy | Number of live births x 100,000 | SIVEP;  SINASC |
| **Perinatal Mortality Rate (per 1,000 births)** | Number of fetal deaths at or after 22 weeks of gestation + Number of neonatal deaths within the first 7 days of life | (Number of fetal deaths at or after 22 weeks of gestation + Number of live births) x 1,000 | SIM;  SINASC |
| **Facility births rate (%)** | Number of women who gave birth in a health facility | Total number of live births x 100 | SINASC |
| **Cesarean Section Rate (%)** | Number of cesarean deliveries (of fetal deaths at or after 22 weeks of gestation plus live births) | Total number of deliveries (of fetal deaths at or after 22 weeks of gestation plus live births) x 100 | SIM;  SINASC |
| **Low Birth Weight Rate (%)** | Number of live births weighing less than 2,500 grams | Total number of live births x 100 | SINASC |
| **Preterm Birth Rate (%)** | Number of live births delivered before 37 weeks of gestation | Total number of live births x 100 | SINASC |
| **Apgar Score < 7 at the 1st Minute Rate (%)** | Number of live births with Apgar scores less than 7 at 1st minute | Total number of live births x 100 | SINASC |
| **Apgar Score < 7 at the 5th Minute Rate (%)** | Number of live births with Apgar scores less than 7 at 5th minute | Total number of live births x 100 | SINASC |

^a^ The numerator and denominator contain population data from the same place (Brazil) and period.

^b^ SIM = Mortality Information System; SINASC = Live Birth Information System; SIVEP = Acute Respiratory Syndromes Epidemiological Surveillance System.

**Supplementary Table 2.** Terminologies and definitions adopted for the study “Maternal and Perinatal Health Indicators in Brazil over a decade: Assessing the Impact of the COVID-19 Pandemic and SARS-CoV-2 Vaccination through Interrupted Time Series Analysis”.

| **Terminology** | **Definition** | **Reference** |
| --- | --- | --- |
| **Maternal death** | The death of a woman during pregnancy or within 42 days of its termination, irrespective of the duration or location of the pregnancy. It includes any causes of deaths related to or aggravated by pregnancy, however, it excludes accidental or incidental causes. | World Health Organization. Maternal and perinatal death surveillance and response: materials to support implementation. Geneva, 2021 |
| **Acute respiratory distress (ARD) during pregnancy** | ARD during pregnancy, consistent with a respiratory viral infection, refers to a condition where pregnant women experience flu-like symptoms such as fever and cough, along with sudden respiratory challenges (dyspnea, cyanosis, low blood pressure, oxygen saturation levels below 95%), regardless of the etiological agent. | Brasil. Ministério da Saúde, Secretaria de Vigilância em Saúde, Departamento de Vigilância das Doenças Transmissíveis. Protocolo de tratamento de Influenza: 2017 [recurso eletrônico]. Brasília, 2018. 49 p.: il. |
| **Perinatal deaths** | It encompasses fetal deaths (at or after 22 weeks of gestation and/or 500g in weight), and early neonatal deaths (within the first 7 days after birth). | World Health Organization. Neonatal and perinatal mortality: country, regional and global estimates. Geneva, 2006. ISBN 92 4 156320 6 |
| **Facility birth** | The process of childbirth occurring in a health facility irrespective of the type of attendant or the place of delivery within the facility | World Health Organization. Institutional births. The Global Health Observatory. Geneva, 2023. Available at https://www.who.int/data/gho/indicator-metadata-registry/imr-details/institutional-birth |
| **Live birth** | The complete expulsion or extraction of a product of conception, where the newborn breathes or shows any other sign of life, such as a heartbeat, umbilical cord pulsation, or voluntary muscle contraction. This can occur with or without cutting the umbilical cord, or detachment from the placenta, and is independent of the duration of pregnancy. | World Health Organization. Neonatal and perinatal mortality: country, regional and global estimates. Geneva, 2006. ISBN 92 4 156320 6 |
| **Low birth weight** | Weight under 2500g at birth, regardless of the duration of gestation. | World Health Organization. Improving maternal and newborn health and survival and reducing stillbirth: progress report 2023. Geneva, 2023. |
| **Preterm newborn** | An infant who is born before completing 37 weeks of gestation | World Health Organization. Improving maternal and newborn health and survival and reducing stillbirth: progress report 2023. Geneva, 2023. |
| **Apgar Score** | The Apgar Score assesses a newborn's status right after delivery or following resuscitation, if required. It gauges five factors: skin color, heart rate, reflexes, muscle tone, and breathing. A score between 7 to 10 is considered favorable, signifying a healthy condition, whereas a score below 7 suggests potential hemodynamic issues. | Watterberg K. L., Aucott S., Benitz W. E., et. al. The Apgar Score. American Academy of Pediatrics Committee on Fetus and Newborn, American College of Obstetricians and Gynecologists Committee on Obstetric Practice. Pediatrics, 2015; 136 (4): 819–822. 10.1542/peds.2015-2651 |

**Supplementary Table 3.** Maternal and perinatal health indicators, according to year of occurrence. Brazil and major regions (North, Northeast, Southeast, South, Central-West), 2013-2022.

|  |  |  |  |  |  |  |  |  |  |  |  |  |
| --- | --- | --- | --- | --- | --- | --- | --- | --- | --- | --- | --- | --- |
| **Variable** | |  | **Year of occurrence** | | | | | | | | | |
| **Local** | |  | **2013** | **2014** | **2015** | **2016** | **2017** | **2018** | **2019** | **2020** | **2021** | **2022** |
| **Maternal mortality ratio (per 100,000 live births)** | | | | |  |  |  |  |  |  |  |  |
|  | North |  | 74,06 | 78,65 | 66,68 | 72,51 | 76,76 | 72,05 | 74,28 | 94,49 | 141,58 | 75,39 |
|  | Northeast |  | 75,11 | 71,30 | 68,53 | 67,58 | 65,83 | 62,74 | 59,36 | 85,90 | 109,39 | 60,65 |
|  | Southeast |  | 49,58 | 51,57 | 52,75 | 53,66 | 58,17 | 52,83 | 52,77 | 65,09 | 104,48 | 47,87 |
|  | South |  | 34,89 | 37,58 | 40,83 | 40,07 | 35,97 | 36,88 | 38,07 | 43,21 | 106,08 | 37,52 |
|  | Central-West |  | 56,67 | 54,27 | 59,37 | 62,59 | 52,03 | 61,38 | 56,41 | 74,19 | 137,11 | 52,60 |
|  | Brazil |  | 58,06 | 58,37 | 57,59 | 58,44 | 58,76 | 56,30 | 55,31 | 71,97 | 113,18 | 53,48 |
|  |  |  |  |  |  |  |  |  |  |  |  |  |
| **Acute respiratory distress during pregnancy (per 100,000 live births)** | | | | | | |  |  |  |  |  |  |
|  | North |  | 26,49 | 12,12 | 5,61 | 20,49 | 8,95 | 10,02 | 14,98 | 390,21 | 504,91 | 107,90 |
|  | Northeast |  | 8,52 | 4,08 | 2,60 | 13,31 | 6,97 | 23,06 | 22,73 | 385,76 | 462,62 | 110,30 |
|  | Southeast |  | 60,12 | 26,97 | 10,37 | 76,63 | 34,81 | 44,46 | 41,98 | 394,53 | 623,63 | 239,57 |
|  | South |  | 69,51 | 24,72 | 19,43 | 88,06 | 40,49 | 58,61 | 59,31 | 297,91 | 731,84 | 514,76 |
|  | Central-West |  | 30,68 | 25,30 | 28,27 | 63,44 | 34,41 | 74,80 | 63,05 | 485,96 | 805,21 | 260,33 |
|  | Brazil |  | 40,77 | 18,53 | 10,37 | 53,43 | 25,00 | 39,08 | 37,70 | 386,02 | 594,04 | 229,40 |
|  |  |  |  |  |  |  |  |  |  |  |  |  |
| **Perinatal mortality rate (per 1,000 births)** | | | |  |  |  |  |  |  |  |  |  |
|  | North |  | 19,36 | 18,95 | 19,12 | 19,04 | 19,03 | 19,12 | 18,84 | 19,25 | 19,55 | 18,67 |
|  | Northeast |  | 21,85 | 20,79 | 21,15 | 20,29 | 20,20 | 19,60 | 19,04 | 19,58 | 19,46 | 19,31 |
|  | Southeast |  | 16,03 | 16,08 | 15,84 | 15,82 | 15,55 | 15,24 | 15,43 | 15,39 | 15,89 | 15,73 |
|  | South |  | 13,76 | 13,95 | 13,70 | 13,06 | 13,27 | 13,01 | 13,12 | 12,59 | 13,04 | 13,30 |
|  | Central-West |  | 16,71 | 15,89 | 16,27 | 15,97 | 15,68 | 15,83 | 15,31 | 15,99 | 16,71 | 16,94 |
|  | Brazil |  | 17,79 | 17,41 | 17,43 | 17,05 | 16,93 | 16,65 | 16,51 | 16,67 | 17,02 | 16,82 |
|  |  |  |  |  |  |  |  |  |  |  |  |  |
| **Facility births rate (%)** | |  |  |  |  |  |  |  |  |  |  |  |
|  | North |  | 95,74 | 95,68 | 96,07 | 95,82 | 95,94 | 96,02 | 96,23 | 95,74 | 95,81 | 96,31 |
|  | Northeast |  | 99,16 | 99,23 | 99,26 | 99,26 | 99,26 | 99,34 | 99,31 | 99,23 | 99,18 | 99,19 |
|  | Southeast |  | 99,68 | 99,65 | 99,62 | 99,59 | 99,59 | 99,56 | 99,52 | 99,49 | 99,43 | 99,39 |
|  | South |  | 99,73 | 99,70 | 99,69 | 99,63 | 99,62 | 99,60 | 99,58 | 99,52 | 99,44 | 99,43 |
|  | Central-West |  | 99,31 | 99,36 | 99,39 | 99,34 | 99,30 | 99,33 | 99,29 | 99,19 | 99,11 | 99,10 |
|  | Brazil |  | 99,09 | 99,09 | 99,13 | 99,08 | 99,09 | 99,10 | 99,09 | 98,98 | 98,91 | 98,97 |
|  |  |  |  |  |  |  |  |  |  |  |  |  |
| **Cesarean section rate (%)** | |  |  |  |  |  |  |  |  |  |  |  |
|  | North |  | 45,79 | 46,58 | 46,01 | 45,33 | 46,34 | 46,96 | 47,54 | 48,47 | 48,95 | 50,83 |
|  | Northeast |  | 49,43 | 50,59 | 49,30 | 49,56 | 49,79 | 51,24 | 52,09 | 52,93 | 53,23 | 55,03 |
|  | Southeast |  | 60,98 | 60,71 | 58,84 | 58,47 | 58,34 | 58,06 | 58,22 | 58,85 | 58,16 | 58,96 |
|  | South |  | 62,45 | 62,20 | 60,29 | 60,49 | 61,17 | 60,78 | 61,12 | 62,61 | 62,44 | 62,15 |
|  | Central-West |  | 61,82 | 62,54 | 61,23 | 61,75 | 62,38 | 62,73 | 62,20 | 63,51 | 63,53 | 63,59 |
|  | Brazil |  | 56,33 | 56,69 | 55,18 | 55,11 | 55,38 | 55,67 | 56,04 | 56,94 | 56,72 | 57,81 |
|  |  |  |  |  |  |  |  |  |  |  |  |  |
| **Low birth weight rate (%)** | |  |  |  |  |  |  |  |  |  |  |  |
|  | North |  | 7,59 | 7,40 | 7,53 | 7,51 | 7,50 | 7,60 | 7,75 | 7,73 | 8,12 | 8,57 |
|  | Northeast |  | 7,94 | 7,73 | 7,91 | 7,93 | 7,99 | 7,90 | 8,19 | 8,15 | 8,41 | 8,95 |
|  | Southeast |  | 9,20 | 9,06 | 9,05 | 9,14 | 9,13 | 9,16 | 9,33 | 9,12 | 9,52 | 10,08 |
|  | South |  | 8,57 | 8,62 | 8,56 | 8,63 | 8,62 | 8,63 | 8,85 | 8,67 | 8,91 | 9,37 |
|  | Central-West |  | 8,26 | 8,20 | 8,29 | 8,26 | 8,25 | 8,44 | 8,51 | 8,54 | 8,92 | 9,47 |
|  | Brazil |  | 8,51 | 8,38 | 8,44 | 8,48 | 8,49 | 8,50 | 8,70 | 8,58 | 8,91 | 9,45 |
|  |  |  |  |  |  |  |  |  |  |  |  |  |
| **Preterm birth rate (%)** | |  |  |  |  |  |  |  |  |  |  |  |
|  | North |  | 12,11 | 11,81 | 11,44 | 11,49 | 11,38 | 11,60 | 12,06 | 11,90 | 12,00 | 12,48 |
|  | Northeast |  | 11,33 | 11,13 | 10,88 | 11,28 | 10,93 | 10,78 | 10,75 | 11,25 | 11,16 | 11,84 |
|  | Southeast |  | 11,70 | 11,22 | 10,75 | 11,01 | 10,92 | 10,99 | 11,05 | 11,24 | 11,30 | 11,72 |
|  | South |  | 10,97 | 10,86 | 10,57 | 11,00 | 10,84 | 10,97 | 11,13 | 11,23 | 11,35 | 11,65 |
|  | Central-West |  | 10,96 | 10,80 | 10,69 | 10,73 | 10,47 | 10,94 | 11,01 | 11,16 | 11,39 | 11,93 |
|  | Brazil |  | 11,48 | 11,18 | 10,83 | 11,12 | 10,92 | 10,99 | 11,09 | 11,31 | 11,35 | 11,85 |
|  |  |  |  |  |  |  |  |  |  |  |  |  |
| **Apgar score < 7 at the 1st minute (%)** | | | |  |  |  |  |  |  |  |  |  |
|  | North |  | 13,30 | 11,92 | 11,19 | 10,90 | 10,84 | 10,36 | 10,51 | 10,20 | 10,07 | 10,02 |
|  | Northeast |  | 14,17 | 14,04 | 13,48 | 13,07 | 12,66 | 12,29 | 12,28 | 12,09 | 12,23 | 12,35 |
|  | Southeast |  | 11,85 | 11,81 | 11,71 | 11,54 | 11,39 | 11,07 | 11,17 | 11,37 | 11,48 | 11,45 |
|  | South |  | 11,86 | 11,90 | 12,11 | 11,96 | 11,94 | 11,88 | 12,16 | 12,03 | 12,55 | 12,67 |
|  | Central-West |  | 12,05 | 11,96 | 11,50 | 11,73 | 11,07 | 11,15 | 11,21 | 10,96 | 11,09 | 11,52 |
|  | Brazil |  | 12,68 | 12,47 | 12,19 | 11,97 | 11,73 | 11,46 | 11,55 | 11,50 | 11,64 | 11,71 |
| **Apgar score < 7 at the 5th minute rate (%)** | | | |  |  |  |  |  |  |  |  |  |
|  | North |  | 2,20 | 2,06 | 2,06 | 2,01 | 2,01 | 1,93 | 1,91 | 1,88 | 1,95 | 1,92 |
|  | Northeast |  | 2,81 | 2,74 | 2,59 | 2,56 | 2,49 | 2,30 | 2,27 | 2,20 | 2,28 | 2,32 |
|  | Southeast |  | 2,13 | 2,13 | 2,11 | 2,07 | 2,02 | 1,97 | 1,97 | 1,97 | 2,01 | 2,00 |
|  | South |  | 2,16 | 2,23 | 2,21 | 2,18 | 2,16 | 2,17 | 2,24 | 2,14 | 2,24 | 2,28 |
|  | Central-West |  | 2,29 | 2,16 | 2,09 | 2,02 | 1,91 | 1,89 | 1,90 | 1,92 | 1,98 | 1,96 |
|  | Brazil |  | 2,35 | 2,31 | 2,25 | 2,21 | 2,16 | 2,08 | 2,08 | 2,05 | 2,11 | 2,12 |
|  |  |  |  |  |  |  |  |  |  |  |  |  |

Sources: Sources: Brazilian Ministry of Health. Live Birth Information System (SINASC), Mortality Information System (SIM), Acute Respiratory Syndrome Surveillance System (SIVEP). Data extracted on 01/25/2024.

**STROBE checklist** for the study titled “Maternal and Perinatal Health Indicators in Brazil over a decade: Assessing the Impact of the COVID-19 Pandemic and SARS-CoV-2 Vaccination through Interrupted Time Series Analysis”.

|  | Item No. | Recommendation |  | Relevant text from manuscript | |  |
| --- | --- | --- | --- | --- | --- | --- |
| **Title and abstract** | 1 | (*a*) Indicate the study’s design with a commonly used term in the title or the abstract |  | Pg 1; lines 2-4 | |  |
|  |  | (*b*) Provide in the abstract an informative and balanced summary of what was done and what was found |  | Pg 2; lines 41 - 62 | |  |
| Introduction | | | | |  | |
| Background/rationale | 2 | Explain the scientific background and rationale for the investigation being reported |  | Pg 3; lines 73 – 77, 83, 88-90, 91-92, 103-107 | |  |
| Objectives | 3 | State specific objectives, including any prespecified hypotheses |  | Pg 4; lines 108-111 | |  |
| Methods | | | | |  | |
| Study design | 4 | Present key elements of study design early in the paper |  | Pg 4; lines 115-118 | |  |
| Setting | 5 | Describe the setting, locations, and relevant dates, including periods of recruitment, exposure, follow-up, and data collection |  | Pg 4; lines 115-118  Pg 5; lines 131-140 | |  |
| Participants | 6 | (*a*) *Cohort study*—Give the eligibility criteria, and the sources and methods of selection of participants. Describe methods of follow-up  *Case-control study*—Give the eligibility criteria, and the sources and methods of case ascertainment and control selection. Give the rationale for the choice of cases and controls  *Cross-sectional study*—Give the eligibility criteria, and the sources and methods of selection of participants |  | Pg 5; lines 138-140  Pg 6; lines 159-160 | |  |
|  |  | (*b*) *Cohort study*—For matched studies, give matching criteria and number of exposed and unexposed  *Case-control study*—For matched studies, give matching criteria and the number of controls per case |  | Not applicable. This is not a matched study. | |  |
| Variables | 7 | Clearly define all outcomes, exposures, predictors, potential confounders, and effect modifiers. Give diagnostic criteria, if applicable |  | Pg 4; lines 115-118  Pg 5-6; lines 141-150  Pg 7; lines 167-176 | |  |
| Data sources/ measurement | 8* | For each variable of interest, give sources of data and details of methods of assessment (measurement). Describe comparability of assessment methods if there is more than one group |  | Pg 6; lines 146-147, 148-150 | |  |
| Bias | 9 | Describe any efforts to address potential sources of bias |  | Pg 8; lines 200-215 | |  |
| Study size | 10 | Explain how the study size was arrived at |  | Not applicable. No samples were calculated, as this study has a national scope and relies on census data. | |  |

Continued on next page

| Quantitative variables | 11 | Explain how quantitative variables were handled in the analyses. If applicable, describe which groupings were chosen and why |  | Pg 6; lines 146-147 |  |
| --- | --- | --- | --- | --- | --- |
| Statistical methods | 12 | (*a*) Describe all statistical methods, including those used to control for confounding |  | Pg 7-9; lines 167-222 |  |
|  |  | (*b*) Describe any methods used to examine subgroups and interactions |  | Not applicable. We did not work with subgroups in this study. |  |
|  |  | (*c*) Explain how missing data were addressed |  | Not applicable. We did not analyze individual participant characteristics as we worked with aggregated data. We also do not have information about missing data since we did not work with individualized data. |  |
|  |  | (*d*) *Cohort study*—If applicable, explain how loss to follow-up was addressed  *Case-control study*—If applicable, explain how matching of cases and controls was addressed  *Cross-sectional study*—If applicable, describe analytical methods taking account of sampling strategy |  | Not applicable. As an interrupted time series study, we worked with aggregated data. There was no individual participant follow-up. No samples were calculated, as this study has a national scope and relies on census data. |  |
|  |  | (*e*) Describe any sensitivity analyses |  | Not applicable. No sensitivity tests were applied in our analyses. |  |
| Results | | | | | |
| Participants | 13* | (a) Report numbers of individuals at each stage of study—eg numbers potentially eligible, examined for eligibility, confirmed eligible, included in the study, completing follow-up, and analysed |  | Not applicable. As previously mentioned, this is a study conducted with aggregated data, focusing on maternal and perinatal health indicators. In Supplementary Material 4, we provide detailed values for all the indicators addressed in this study, on an annual basis, and stratified by macro-regions of Brazil. |  |
|  |  | (b) Give reasons for non-participation at each stage |  | Not applicable. All records of births, fetal (≥20 gestational weeks), early neonatal (<7 days of life) and maternal deaths, and cases of acute respiratory distress in pregnant women were eligible, and used in the calculations of the indicators. |  |
|  |  | (c) Consider use of a flow diagram |  | Not applicable. |  |
| Descriptive data | 14* | (a) Give characteristics of study participants (eg demographic, clinical, social) and information on exposures and potential confounders |  | Not applicable. As previously mentioned, this is a study conducted with aggregated data about births, fetal (≥20 gestational weeks), early neonatal (<7 days of life) and maternal deaths, and cases of acute respiratory distress in pregnant women, focusing on maternal and perinatal health indicators. Individual participant data were not accessed. |  |
|  |  | (b) Indicate number of participants with missing data for each variable of interest |  | Not applicable. As previously mentioned, this is a study conducted with aggregated data about births, fetal (≥20 gestational weeks), early neonatal (<7 days of life) and maternal deaths, and cases of acute respiratory distress in pregnant women, focusing on maternal and perinatal health indicators. Individual participant data were not accessed. |  |
|  |  | (c) *Cohort study*—Summarise follow-up time (eg, average and total amount) |  | Not applicable. As previously mentioned, this is a study conducted with aggregated data about births, fetal (≥20 gestational weeks), early neonatal (<7 days of life) and maternal deaths, and cases of acute respiratory distress in pregnant women, focusing on maternal and perinatal health indicators. Individual participant data were not accessed. |  |
| Outcome data | 15* | *Cohort study*—Report numbers of outcome events or summary measures over time |  | Not applicable. This is an interrupted time series study. The detailed values for studied indicators, by year of occurrence, are available in Supplementary Material 4. |  |
|  |  | *Case-control study—*Report numbers in each exposure category, or summary measures of exposure |  | Not applicable. This is an interrupted time series study. The detailed values for studied indicators, by year of occurrence, are available in Supplementary Material 4. |  |
|  |  | *Cross-sectional study—*Report numbers of outcome events or summary measures |  | Not applicable. This is an interrupted time series study. The detailed values for studied indicators, by year of occurrence, are available in Supplementary Material 4. |  |
| Main results | 16 | (*a*) Give unadjusted estimates and, if applicable, confounder-adjusted estimates and their precision (eg, 95% confidence interval). Make clear which confounders were adjusted for and why they were included |  | All values obtained in the interrupted time series analysis were presented in **Tables 1 and 2**, along with their respective 95% confidence intervals. |  |
|  |  | (*b*) Report category boundaries when continuous variables were categorized |  | Not applicable. We did not work with categorized continuous variables in this study. |  |
|  |  | (*c*) If relevant, consider translating estimates of relative risk into absolute risk for a meaningful time period |  | Not applicable. We did not work with measures of relative risk in this study. |  |

Continued on next page

| Other analyses | 17 | Report other analyses done—eg analyses of subgroups and interactions, and sensitivity analyses |  | Not applicable. There were no subgroup or interaction analyses, and no sensitivity tests in this study. |
| --- | --- | --- | --- | --- |
| Discussion | | | | |
| Key results | 18 | Summarise key results with reference to study objectives |  | Pg 11-12; lines 272-286  Pg 13; lines 317-323  Pg 14; lines 345-351 |
| Limitations | 19 | Discuss limitations of the study, taking into account sources of potential bias or imprecision. Discuss both direction and magnitude of any potential bias |  | Pg 14-15; lines 352 – 360, 367-373 |
| Interpretation | 20 | Give a cautious overall interpretation of results considering objectives, limitations, multiplicity of analyses, results from similar studies, and other relevant evidence |  | Pg 14-15; lines 352-373 |
| Generalisability | 21 | Discuss the generalisability (external validity) of the study results |  | Pg 14-15; lines 352-373 |
| Other information | |  | | |
| Funding | 22 | Give the source of funding and the role of the funders for the present study and, if applicable, for the original study on which the present article is based |  | Pg 16-17; lines 403-410 |

**Note:** Information on the STROBE Initiative is available at www.strobe-statement.org.
